# Aptamer- Based Label-Free Electrochemical Biosensor Array for the Detection of Total and Glycated Hemoglobin in Human Whole Blood

**DOI:** 10.1038/s41598-017-01226-0

**Published:** 2017-04-21

**Authors:** Shimaa Eissa, Mohammed Zourob

**Affiliations:** 1 Department of Chemistry, Alfaisal University, Al Zahrawi Street, Al Maather, Al Takhassusi Road, Riyadh, 11533 Saudi Arabia; 2 King Faisal Specialist Hospital and Research Center, Zahrawi Street, Al Maather, Riyadh, 12713 Saudi Arabia

## Abstract

The increase of the level of glucose in blood leads to an increase in the fraction of glycated hemoglobin (HbA1c). Therefore, the percentage of HbA1c in the blood can serve as a marker for the average glucose level over the past three months and thus, it can be used to diagnose diabetes. Here, we report the selection, identification and characterization of specific DNA aptamers against HbA1c- and total hemoglobin (tHb) and their integration into an electrochemical array sensing platform. High affinity and specificity aptamers were selected *in vitro* showing dissociation constants of 2.8 and 2.7 nM for HbA1c and tHb, respectively. Thiol-modified forms of the aptamers were then immobilised on gold nanoparticles (AuNPs)-modified array electrodes and used for the label-free detection of HbA1c and tHb using square wave voltammetry. The voltammetric aptasensors showed high sensitivity with detection limits of 0.2 and 0.34 ng/ml for HbA1c and tHb, respectively. This array platform is superior to the currently available immunoassays in terms of simplicity, stability, ease of use, reduction of sample volume and low cost. Moreover, this method enabled the detection of HbA1c % in human whole blood without any pre-treatment, suggesting great promise of this platform for the diagnosis of diabetes.

## Introduction

Diabetes mellitus is a life-long metabolic disease that can cause several complications representing one of the most important health concerns nowadays. The early diagnosis of diabetes and the regular examination of blood glucose level are essential factors in preventing the health complications resulting from this disease.

In the normal 120-day lifespan of the red blood cell, glucose molecules react with hemoglobin, accumulating an adduct known as glycated hemoglobin. Glycated hemoglobin is an adduct that results from the non-enzymatic reaction of glucose with the N-terminal valine of hemoglobin β-chains^1^. The ratio of glycated hemoglobin to the total hemoglobin within the red blood cell, therefore, reflects the average level of glucose to which the cell has been exposed during its life-cycle, and can be used a marker for average blood glucose over the previous months prior to the measurement^2^. In contrast to the common plasma glucose tests, the level of glycated hemoglobin is not affected by daily fluctuations in the blood glucose concentration but reflect the average glucose levels over the prior six to eight weeks^3^.

Glycated hemoglobin testing is recommended for checking blood sugar in people who might be pre-diabetic. In fact, in 2010 American Diabetes Association (ADA) added the blood concentration of glycated hemoglobin (HbA1c) over 6.5% as another criterion for the diagnosis of diabetes^4, 5^. Screening of elevated HbA1c level to a broader population represents an effective way for early diagnosis of diabetes. Higher amounts of HbA1c not only indicate poorer control of blood glucose levels, but also associates with cardiovascular disease, nephropathy, and retinopathy, emphasizing the importance of the precise and accurate monitoring of the percentage of HbA1c. Furthermore, monitoring the HbA1c in type-1 diabetic patients may improve treatment^5^.

Current methodologies for HbA1c detection are mainly based on either charge differences (chromatography)^6^, structure (affinity or immunoassay assays) or enzymatic assays with the aim to differentiate between HbA1c and native Hb. According to the results of the GH-2 survey of the HbA1c test done by the College of American Pathologists (CAP, USA), immunoassays are the most commonly used methods (65% of participants) followed by the cation-exchange chromatography (31%) then the affinity chromatography (4%)^7^. In the chromatography-based methods, the HbA1c% is photometric determined by measuring the ratio of the HbA1c peak area over the tHb peak area. Thus, false positive or negative results can be obtained using these methods due to the possible interferents from the blood^8^. Moreover, these methods are generally performed in centralized laboratories using large and expensive instruments. Several immunoassays have been used for the quantification of HbA1c% using specific mono-or polyclonal antibodies to HbA1c^9^. Separate ELISA kits based on sandwich assays for the detection of both HbA1c and tHb are commercially available. However, these kits are not intended to produce results for clinical use and cannot be accurately utilised for HbA1c% detection. Immunoassays based on field effect transistor^10^ or electrochemical detection using boric acid-modified electrode have been reported^11^. However, the borate-modified electrodes can recognise the blood albumin causing interference. Some sandwich assays using specific HbA1c antibody as capture probe and lectin or glycan-binding antibodies as detection probe have been reported^12^. However, these methods have low sensitivity due to the interference from other glycan moieties in the blood sample and the high background signal. In other study, a polyclonal antibody against Hb was used as a common capture probe which binds to all forms of Hb and specific monoclonal antibodies against tHb and HbA1c were used as detection probes^13^. This immunosensor fabricated using array system has eliminated the use of glycan binding molecules and thus, significantly reduced the background interference, achieving high sensitivity. However, immunoassays in general suffer from the instability of the antibodies, their high cost, batch-to-batch variations which limits the clinical usefulness of these methods^14^. Therefore, the development of low cost, stable, portable, specific and simple biosensing platform for the detection of HbA1c is highly demanded, and would facilitate the routine monitoring of HbA1c % in blood for the early diagnosis of diabetic patients.

Aptamers are short single stranded DNA (ssDNA) or RNA sequences that have been recently appeared^15^ as novel recognition receptors which can be used as alternative to antibodies in biosensing devices. Aptamers can be selected *in vitro* against a variety of targets including small molecules, metal ions and proteins using a process known as SELEX^16^. Because of their high affinity and stability, low-cost and ease of synthesis with high reproducibility, DNA aptamers are being used as recognition elements replacing antibodies in many biosensing platforms. Recently, Lin *et al*.^17^ have reported the first identification of specific aptamers against HbA1c and tHb using microfluidic SELEX chip from a randomized 40-mer DNA library. In this report, the authors have shown a preliminary application of the selected aptamers in an aptamer-antibody sandwich -like chemiluminescence immunoassay. However, no biosensing application of the selected aptamers has been reported so far. We believe that the selection of other aptamer sequences for Hb and HbA1c gives diversity in applying different aptamers that have different molecular structures in a variety of biosensing platforms^18^. Here, we report the selection, identification and characterization of new DNA aptamers against HbA1c and tHb from a 60-mer library. The integration of the new aptamers in a label-free voltammetric array biosensor platform was presented for the first time. The proposed platform offers several advantage over current technologies: (1) the low cost of the aptamers and the array screen printed electrodes reduce the overall cost of the device, (2) the long term stability of the biosensor due to the use of the DNA aptamers, (3) only microlitters of blood samples are used for analysis, (4) electrochemical transducers are easy to use and can be miniaturized^19–22^ and (5) the label-free detection format helps to reduce the cost and maintain the affinity of the aptamers. Because of these advantages, we believe that this new HbA1c array platform will facilitate the use of this test by both patients and healthcare professionals at home or small clinics and can be further developed for point-of-care (POC) diagnostics. The HbA1c POC diagnostic device will allow a broader screening of diabetes and will help in the early diagnosis and management.

## Results and Discussion

### Immobilization of hemoglobin and glycated hemoglobin-A1C on the NHS-activated sepharose beads

The immobilization of target proteins on solid matrix is essential step in order to separate the bound from unbound DNA sequences during the SELEX process. Here, we coupled Hb and HbA1c proteins to commercial sepharose beads via the reaction of the NHS terminal of the beads with the amine groups of the proteins (Fig. S1). Then, the coupling reaction was verified using direct ELISA. The blue color observed in the HbA1c-beads indicated the success of the covalent immobilization(Fig. S2).

### *In Vitro* Selection of the DNA aptamers against HbA1c

A SELEX protocol was performed for the selection of specific aptamers to *HbA1c*. The aptamers should specifically recognise the glucose-bound amino acids at the N-terminal of the β- chain of Hb in order to have the capability to distinguish the glycated from the non glycated Hb. The selection of the aptamers was performed following the protocol that we reported previously^23–25^. Briefly, a DNA library consisting of 1.8 × 10^15^ random sequences is incubated with the HbA1c-beads, followed by a separation step of the beads-bound DNA from the unbound by washing with binding buffer. Then the bound DNA is nonspecifically eluted by denaturation using urea and heating. To enrich the bound DNA sequences, the eluted DNA pool is amplified by PCR after desalting to remove the urea. We used a fluorescent labelled primer to enable the separation of the fluorescent ssDNA aptamer from the PCR product via denaturing PAGE by visualising the fluorescent band and also to facilitate the quantification of the eluted DNA. This cycle of binding, partitioning, amplification and purification is repeated several times to obtain the highest affinity aptamers. Two counter selection steps were performed during the SELEX process. The first counter selection using negative blocked NHS activated beads was introduced after the fifth round to eliminate the DNA sequences that bind to the beads matrix. The second counter selection was performed using Hb-beads in order to exclude most of the DNA sequences which bind to Hb and to enable the selection of the HbA1c-specific aptamers. The selected DNA pool from the sixth round was incubated with Hb-beads, followed by washing the beads and incubating the DNA pool, collected from the washes with the positive HbA1c beads. To check the progress of the enrichment of the specific DNA to HbA1c through the selection process, the fluorescence intensity of the eluted DNA from each round was measured. Figure S4 shows the gradual increase in the DNA recovery with increasing the number of rounds. A small drop in the DNA recovery after the first counter selection with negative beads was observed, likely due to the elimination of the sequences that have certain affinity to the sepharose beads. In fact, the DNA recovery was low until the ninth round and a significant increase in the recovery was seen in the last two rounds. This increase suggests the enrichment of the HbA1c-specific DNA. Thus, the DNA pool collected from round 11 was cloned into *E. coli* competent cells. Then, 21 clones were randomly selected for sequencing. Then, PRALINE software was used to analyse the selected sequences by multiple sequence alignment^26^, showing significant sequence convergence which indicates the successful enrichment of the DNA pool from round 11. We then grouped the selected sequences into six different families (A–F), based on their similarities (Fig. S5) and representative aptamers from each group were tested for the binding to Hb and HbA1c.

### Binding affinity studies and determination of dissociation constants of the aptamers-Hb and -HbA1c complexes

A sensitive and simple electrochemical assay was used to study the binding affinity of the selected aptamers to the Hb and HbA1c proteins. For that, both Hb and HbA1c were individually immobilized on gold electrodes.

### Immobilization of Hb and HbA1c on the gold electrodes

Both Hb and HbA1c proteins were immobilized on gold electrodes via the amine groups of the proteins. The gold surface was first modified via the formation of self-assembled monolayer (SAM) of cysteamine. Then, 1,4-phenylene diisothiocyanate which serves as a bifunctional linker was employed to link the amine groups of the proteins to the terminal amine groups of Cys/Au by covalent bond (Fig. S3) forming carbamide moiety. To verify the successful functionalization of the gold electrodes and the attachment of the Hb and HbA1c proteins, cyclic voltammetry (CV) and electrochemical impedance spectroscopy (EIS) were used (Fig. S6). The Impedance spectra were fitted using modified Randles equivalent circuit (Fig. S7).

### Binding affinity study of the selected aptamers

**Figure 1:**
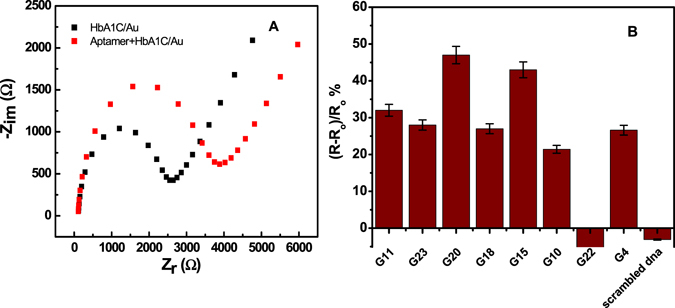
(**A**) Example of Nyquist plots of the HbA1c-modified gold electrodes before (black) and after (red) aptamer binding. (**B**) Binding assays with individual aptamer sequences to HbA1c-modified electrodes.

**Figure 2:**
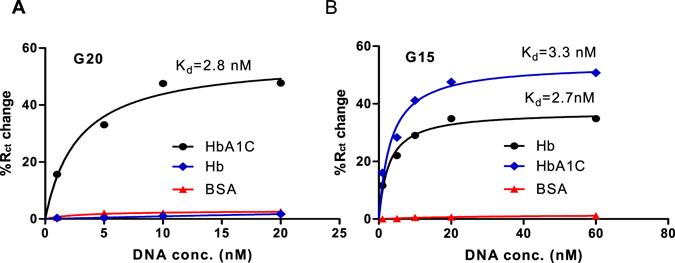
Binding curves of the selected aptamers G20 (**A**) with HbA1c and nonspecific proteins and aptamer G15 (**B**) with Hb, HbA1c and non specific protein.

Eight representative aptamers were subjected to initial binding analysis. Initial screening of the sequences (G11, G23, G20, G18, G15, G10, G22, G4) was performed using EIS to confirm their successful binding to HbA1c (Fig. 1A). As shown in Fig. 1B, a comparative binding analysis was done for the aptamer sequences to HbA1c by monitoring the percentage change of the R_CT_ of HbA1c- modified electrodes after binding with each aptamer. A significant change in the R_CT_ after the incubation of most of the tested aptamers with the HbA1c electrodes was observed, while no response was obtained with scrambled DNA sequence (control DNA), indicating specific binding of the aptamers to HbA1c. Only one sequence (G22) did not show good binding to HbA1c. The highest change in EIS signal was obtained for aptamers G11, G23, G18, G20, G15; therefore these aptamers were subjected to further analysis to assess their binding affinity. The dissociation constants were determined by incubating different concentrations of the aptamers with Hb and HbA1c- modified electrodes. As shown in Fig. 2, binding curves were obtained by plotting the change in the electron transfer resistance versus the aptamers concentrations. The K_d_ were then calculated from the binding curves using non linear regression analysis. As shown in Table 1, two different types of sequences were identified and the K_d_ values of the aptamers were shown to be in the nanomolar range. Four HbA1c -specific aptamers with high affinity and specificity were obtained (G11, G18, G20, G23). It was noticed that G20 exhibited the highest binding affinity to HbA1c (K_d_ = 2.8 nM). However, interestingly, the aptamer sequence G15 showed good binding to both Hb and HbA1c with almost similar K_d_ of 2.7 and 3.3 nM. Thus, from our SELEX screening, an aptamer that bind to the tHb (G15) as well as specific aptamers to HbA1c (G20) were obtained. It is worth mentioning that no significant common motifs between our new selected aptamers and the recently reported aptamers. Moreover, our new aptamers showed higher affinity to Hb and HbA1c than the reported sequences (K_d_ of 7.6 and 7.3 nM for HbA1c and Hb)^17^. In fact, the K_d_s of the reported aptamer sequences were calculated using a magnetic bead-based chemiluminescence assay^17^ which can be different in the accuracy than our impedance affinity assay. Therefore, we expect in the future to study the K_d_s of the old and the new aptamers using same method for accurate comparison of the results.

**Table 1:** Sequences and dissociation constants (*K*_d_) between Hb and HbA1c and selected aptamers.

Clone number	Aptamer sequence	Kd (nM) HbA1c	K_d_ (nM) Hb
G11	CGACACCAGCACACAGACCCGAGACACACGTCAGATCAACAGCGACCGTATCATTGGTTG	108	NB
G18	GGCCACAGCAGCCAGTACACCCACCCACCAGCCCCGTCAACGACCTGAACCTGCCCTGTGTG	95	NB
G15	ACGCACACCAGAGACAAGTAGCCCCCCAAACGCGGCCACGGAACGCAGCACCTCCATGGC	3.3	2.8
G20	GGGGACACAGCAACACACCCACCCACCAGCCCCAGCATCATGCCCATCCGTCGTGTGTG	2.8	NB
G23	GGACACGGCAAAGGGGTATAGCCTACCGGACCGTGGACATGGAATTGTGTGCTGCGTGG	303	NB

The binding specificity of the HbA1c aptamer (G20) was also confirmed by incubating the HbA1c modified electrode with different concentrations of non-glycated Hb and BSA as controls. No significant response was obtained for the non specific proteins which indicates excellent specificity of the selected aptamer (Fig. 2). Similarly, the specificity of the tHb aptamer (G15) was verified against BSA as control. The two aptamers, G15 and G20 were then applied for Hb and HbA1c detection in an array platform.

### Voltammetric array Aptasensors for HbA1c Detection

**Figure 3:**
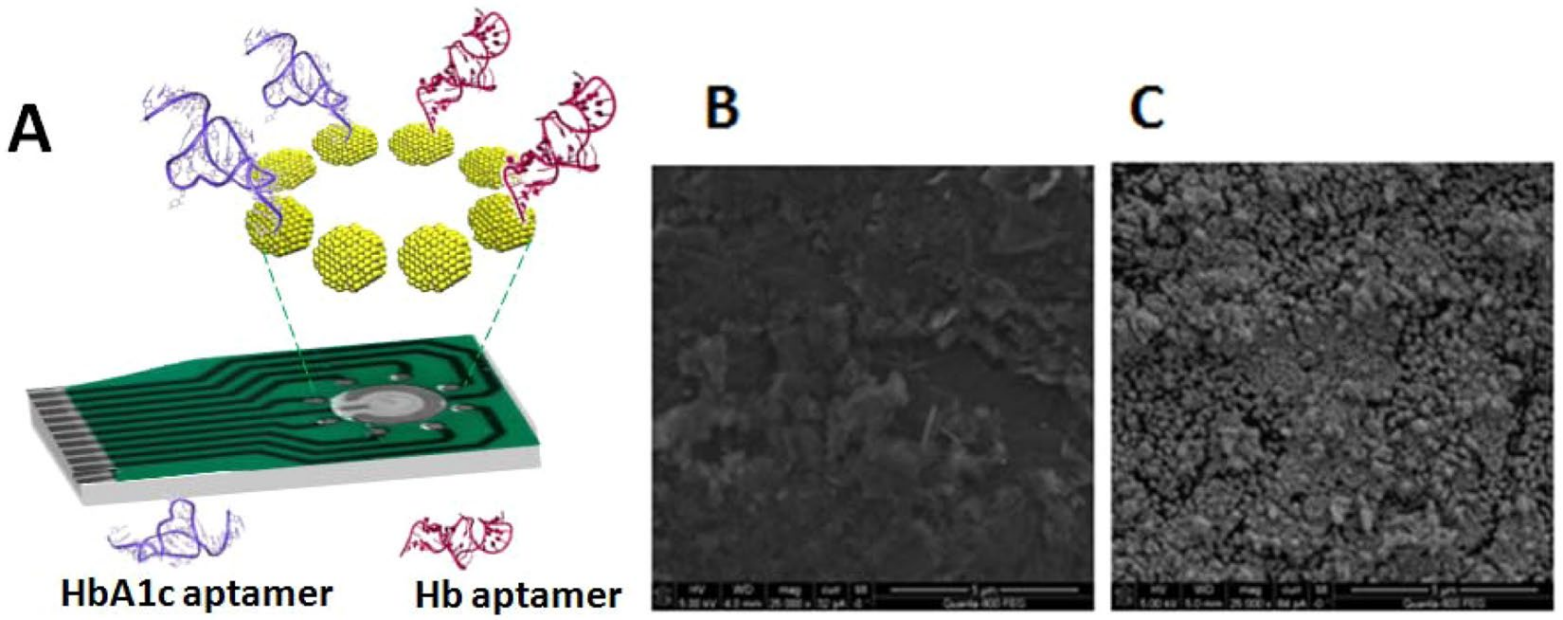
(**A**) Schematic diagram of the aptasensor array platform and scanning electron microscopic image of the screen printed carbon electrodes before (**B**) and after (**C**) AuNPs deposition.

The array screen printed carbon electrodes (Fig. 3A) were first modified by deposition of AuNPs via electroreduction of gold chloride. Figure 3 shows the scanning electron microscopic (SEM) image of the carbon electrode before (A) and after (B) AuNPs deposition. The two aptamers (G15 and G20) were then immobilized on different AuNPs-modified electrodes on the same chip array by self-assembly of the thiol -modified aptamers. After aptamers immobilization, the electrodes were blocked by MCH to form a mixed monolayer with the thiol-modified aptamers. This step was previously shown to be very important to reduce the nonspecific adsorption of the ssDNA on the surface and thus, preserve the conformation of the aptamer^24, 27^.

**Figure 4:**
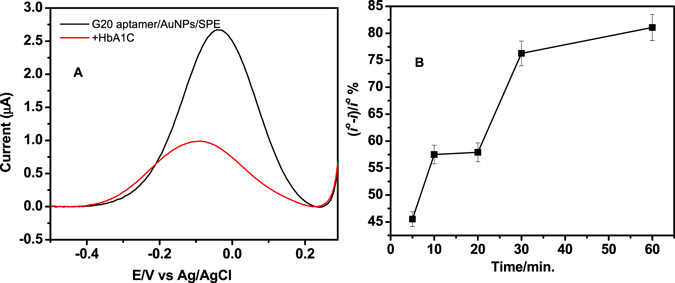
(**A**) Example of square wave voltammetry signal of the aptasensor before (black) and after (red) binding with HbA1c, (**B**) Effect of the binding time on the HbA1c aptasensor response signal.

After the immobilization of the aptamers on the electrodes, square wave voltammetry (SWV) was used to monitor the protein binding by measuring the reduction peak current of [Fe(CN)_6_]^4−/3−^ redox couple. As shown in Fig. 4A, after HbA1c binding, a decrease in the peak current was noticed due to the blocking effect of this bulky protein. This decrease in the SWV current represents the basis of the aptasensor detection signal.

### Binding time optimization of the aptasensors

The aptasensor’s response ((i − i°)/i°%) was measured after incubating 1 µg/ml of tHb and HbA1c on the G15 and G20 modified electrodes, respectively at different time points. As shown in Fig. 4B, the reduction peak current decrease with increasing the incubation time. Maximum signal change was achieved after 30 min incubation with the HbA1c protein. Therefore, 30 min was selected as the binding time in the subsequent experiments for Hb and HbA1c.

### Dose response of the aptasensors

**Figure 5:**
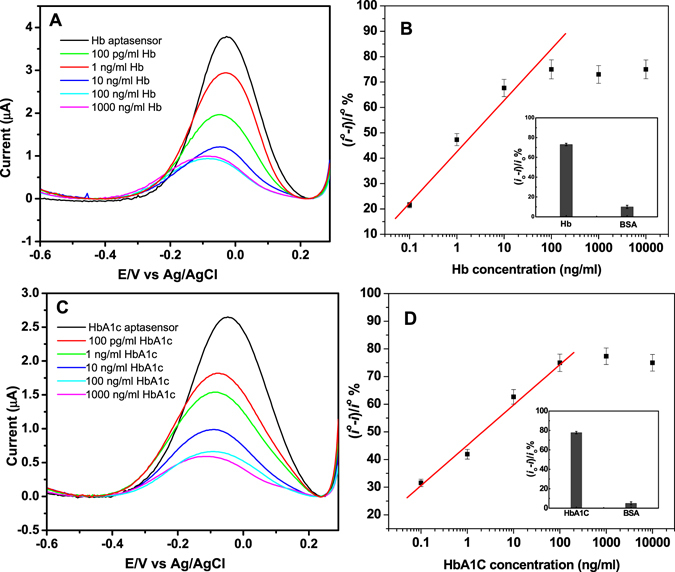
SWVs of the aptasensors before and after binding with different concentrations of Hb (**A**) and HbA1c (**C**), Calibration curves for Hb (**B**) and HbA1c (**D**). Insets are the specificity studies of the aptasensors against BSA.

The aptasensors voltammetric response toward tHb and HbA1c was measured in the concentration range of 100 pg/mL to 10 µg/mL. As shown in Fig. 5A and C, significant drops in the reduction peak current were seen with increasing concentrations of Hb and HbA1c due to the binding of the proteins to their specific aptamers which blocks the electron transfer as explained above. Figure 5 shows the calibration plots (the percentage change in the peak current after the Hb (Fig. 5B) and HbA1c (Fig. 5D) binding vs. the concentration. Three independent measurements were done for each data point in order to assess the reproducibility of the aptasensors array. Both HbA1c and tHb aptasensors showed linear response within a concentration range from 100 pg/mL to 100 ng/mL. The linear regression equation of the HbA1c aptasensor is (*i*° *−* *i)/i*°*%* = 45.0 + 14.6 × log*C* (ng/ml), R = 0.99, with a detection limit (LOD) of 0.2 ng/mL and for the tHb: (*i*° *−* *i)/i*°*%* = 42.5 + 20.2 × log*C* (ng/ml), R = 0.98, with LOD = 0.34 ng/ml. The LOD was calculated from 3(S_y/x_/m), where S_y/x_ is the standard error of estimate and m is the slope of the calibration curve. It is worth noting that, these detection limits are lower than the LOD of the commercial ELISA kits as well as than the reported immunosensor array platform^13^. The selectivity of the aptasensors was also confirmed by incubating the aptasensors with BSA. As shown in insets of Fig. 5, high response signals were observed only when the aptasensors were incubated to their specific protein, while no response was obtained with BSA indicating that no effect of nonspecific adsorption was obtained.

### Detection of HbA1c % in whole blood samples

The proposed aptasensor platform was then tested with four quality control human blood samples in order to validate the assay. As shown in Fig. S8A, the aptasensor response signal for HbA1c decreases as the standard blood sample (LN15-08) was serially diluted from 10^−2^ to 10^−5^. However, for the tHb, the aptasensor response decreases for the diluted samples from 10^−5^ to10^−3^ while no further signal change was observed at the higher concentrated samples (10^−2^). When the concentration of the tHb is high, a saturation of the aptasensor occur. Therefore, 10^−4^ or 10^−3^ should be used for analysis. These results indicate that the proposed aptasensor array platform is able to distinguish and detect tHb and HbA1c over a 3 order of magnitude concentration range. By referring to the calibration curves, the % HbA1c in the sample was calculated to be 6.67% which is in very good agreement with the values given by the college of American pathologist. A linear relationship was also obtained between the % HbA1c and the HbA1c aptasensor response signals within a concentration range of 6.67–10.47% (Fig. S8B). Therefore, it is clear that the developed aptasensor array platform can be applied to discriminate between diabetic and healthy individuals.

## Experimental

The materials and instruments used in this work are described in the supporting information.

### Coupling of HbA1c and tHb to the NHS activated beads

The coupling reaction is shown in the schematic diagram (Fig. S1). Stock solutions of purified Hb (363 µg) and HbA1c (1.9 mg) in coupling buffer (0.1 M NaHCO_3_, 0.5 M NaCl, pH 8.3) were prepared. Two millilitres of the NHS activated beads were washed for 15 min with 1 mM HCL to remove the additives and preserve the activity of the reactive groups. Then, the washed beads were incubated with the stock solution of either Hb or HbA1c (1:1 volume ratio) in polypropylene tubes and mixed end-over-end for 4 hours at 4 °C. After the incubation, the beads were rinsed five times with coupling buffer to eliminate the excess proteins. The unreacted NHS active groups on the beads were then blocked by transferring the beads to 0.1 M Tris-HCL buffer pH 8 and mixing for 1 hour. After that the beads were rinsed extensively with three cycles of alternating pH. Each cycle consists of a wash with 0.1 M acetic acid/sodium acetate, pH 4 containing 0.5 M NaCl followed by a wash with 0.1 M Tris-HCL buffer pH 8 containing 0.5 M NaCl. Simultaneously, negative NHS activated beads were also prepared to be used for the counter selection cycle by blocking the beads with 0.1 M Tris-HCL buffer pH 8. The Hb beads, HbA1c beads and the negative beads were kept in 50 mM Tris-HCl buffer pH 7.5 at 4 °C until further use. The immobilization of Hb and HbA1c on the beads was verified by performing direct ELISA for the coupled beads and the negative beads as control. To perform the ELISA experiments, 30 μl of each beads were rinsed with PBS buffer and blocked with 2% BSA in PBS buffer overnight at 4 °C. Then the beads were incubated with diluted anti-HbA1c antibody (1:1000) in PBS buffer, pH 7.4. After washing, the beads were incubated with HRP-labelled secondary antibody (diluted 1:1000) for 1 h. Then the beads were washed three times with PBS buffer and stabilized chromogen (TMB) solution was added. A blue color was developed in the HbA1c -coated beads, while no color was seen in the Hb coated beads as well as in the negative beads indicating the successfulness of the conjugation reaction (Fig. S2). The stability of the Hb and HbA1c-beads were also confirmed by ELISA after storage for few weeks during the SELEX process.

### *In vitro* selection of the DNA Aptamer

The protocol for the aptamers selection, cloning and sequencing is described in detail in the supporting information.

### Immobilization of Hb and HbA1c on the gold electrode

The polycrystalline Au electrodes were polished with aqueous alumina slurries of 1 mm and 0.05 mm, then rinsed with water. The electrodes were then immersed in fresh Piranha’s solution (1:3 v/v, H_2_O_2_ and H_2_SO_4_) for 2 min. and subsequently washed with Milli-Q water and ethanol. Then, the Au electrodes were subjected to electrochemical cleaning by cyclic voltammetry cycling between 0.20 and 1.6 V vs. Ag/AgCl (3 M KCl) at 100 mV/s in 0.1 M sulphuric acid until the characteristic CV of a clean gold is obtained. After cleaning, the gold electrodes were functionalized as shown in the schematic diagram (Fig. S3). The gold electrodes were incubated in 10 mM cysteamine hydrochloride for 2 hours at room temperature to form self-assembled monolayers. Then, the electrodes were rinsed with water and absolute ethanol to remove unreacted cysteamine residues. The terminal amine groups of the cysteamine-modified gold electrode (Cys/Au) were then incubated with 10 mM PDITC in pyridine and N,N-dimethyl formamide (DMF) (v:v, 1:9) for 2 h. Then, the electrodes were washed with DMF, ethanol and dried. The PDITC-modified electrodes were then incubated in 10 µg/ml of Hb or HbA1c solution in PBS buffer pH 7.4 for 2 h and then rinsed with PBS buffer to remove the unbound proteins. The modified-electrodes were incubated with 1% BSA in PBS buffer for 30 min to deactivate the unreacted thiocyanate groups and block the free gold surface and then extensively washed with PBS buffer. The control aptasensor was prepared by incubating the PDITC-modified electrode with 3% BSA in PBS buffer (pH 7.4) for 2 hours. The Hb and HbA1c-modified electrodes were rinsed with PBS buffer and kept at 4 °C in PBS buffer until further use.

### Binding analysis of the aptamer sequences to Hb and HbA1c

In order to test the binding affinity of the aptamer sequences to their protein targets, some representative sequences were synthesized after eliminating the primers sequences. 25 nM solution from each synthesized sequence in binding buffer was incubated with the Hb and HbA1c-modified electrodes for 30 min. After binding, the electrodes were washed with binding buffer and impedance measurements were performed in 5 mM [Fe(CN)_6_]^3−/4−^ redox couple. The binding was then evaluated by calculating the % R_CT_ change ((R − R^o^)/R^o^%).

### Dissociation constants determination by electrochemical assay

The dissociation constants of the selected aptamers for Hb and HbA1c were determined by performing binding assays as described above using different concentrations of the aptamer (0 to 200 nM). The change in the R_CT_ after binding with each aptamer sequence was measured and saturation curve was obtained for each aptamer. The dissociation constant (K_d_) for each aptamer with HbA1c and Hb was determined by non-linear regression analysis.

### Array electrodes modifications and aptasensors fabrication

The eight carbon working electrodes of the array chip were modified with gold nanoparticles (AuNPs). The chip was covered with 100 µl of 6 mM HAuCl_4_ solution in 0.1 M KNO_3_ and electrodeposition was performed using 20 cyclic voltammetry scans from −0.2 to −1.2 V at 50 mV/s.

For immobilization of aptamers, the thiol-modified Hb aptamer (G15) and HbA1c aptamer (G20) solutions in binding buffer were incubated separately onto different AuNPs-modified electrodes on the array chip for 12 h at water saturated atmosphere. After immobilization, the electrodes were rinsed with binding buffer and incubated with 1 mM MCH in PBS buffer, pH 7.4 for 30 min. The aptasensors were then washed thoroughly with binding buffer and immediately employed in the electrochemical experiments, or stored in binding buffer solution at 4 °C until further use.

### Electrochemical measurements

For the binding affinity studies, EIS was performed. The AC voltage was 10 mV, the frequency range was from 10 kHz to 1.0 Hz and the DC potential was 0.20 V (versus a Ag/AgCl reference electrode). The impedance spectra were presented in the form of Nyquist plots. Nova 1.11 software was used to fit all the impedance spectra. All the impedance measurements were done in 5 mM solution of [Fe(CN)_6_]^3−/4−^ redox pair (1:1 molar ratio) in a 0.1 M PBS buffer, pH 7.4.

For the Hb and HbA1c detection and selectivity experiments, each aptamer-modified electrode on the aptasensor array chip was incubated for 30 min with specific concentrations of Hb and HbA1c standard solutions. The electrodes were then washed with 50 mM Tris-HCl buffer pH 7.4 and subjected to SWV measurements in 10 mM [Fe(CN)_6_]^3−/4−^ redox couple in 0.1 M PBS buffer solution. The parameters used for the SWV measurements: amplitude 20 mV; interval time 0.04 s; step potential −5 mV; scan rate 125 mV s^−1^, and frequency 25 Hz. The CV experiments were conducted at a scan rate of 100 mV/s.

### Application of the aptasensors in standard human whole blood samples

Quality control Samples (LN15-08–LN15-11) of human whole blood obtained from College of American Pathologists were used to validate the aptasensor. The concentrations of HbA1c in the samples are: LN15-08: 6.67%, LN15-09: 7.94%, LN15-10: 9.18% and LN15-11: 10.47%. The four blood samples were serially diluted by orders of magnitude ranging from 10^−2^ to 10^−5^ with deionized water (1 µl of blood was diluted to 100 µl with deionized water and the next dilutions were done in binding buffer). 2 µl of each diluted blood sample were then incubated on different spots of the Hb and HbA1c modified aptasensor array and kept for 30 min at room temperature. Then the electrodes were washed with 50 mM Tris-HCl buffer pH 7.4 and subjected to SWV measurements as previously described.

## Conclusions

High affinity DNA aptamers for HbA1c and tHb were successfully selected using SELEX after 11 rounds of selection. The tested aptamers bind to HbA1c with dissociation constants in the nanomolar range with the highest affinity aptamer, G20, exhibiting a K_d_ of 2.8 nM. Another aptamer sequence which showed high binding affinity to tHb with a K_d_ of 2.7 nM was also selected. The HbA1c and tHb-specific aptamers were then applied for the detection of HbA1c % using a voltammetric aptasensor array platform, showing remarkable sensitivity and selectivity. The aptasensor array platform was validated using standard human whole blood samples and demonstrated linearity over wide concentration range. We believe that the developed platform is superior to current methodologies due to the simplicity, stability and lower cost which will facilitates the early and accurate diagnosis of diabetes.

## Electronic supplementary material

supporting info
