# Supplementary material for: Aptamer- Based Label-Free Electrochemical Biosensor Array for the Detection of Total and Glycated Hemoglobin in Human Whole Blood
Source: Sci Rep. 2017 Apr 21;7:1016. doi: 10.1038/s41598-017-01226-0 (PMC5430690; doi:10.1038/s41598-017-01226-0)
Supplement: Supplementary file 1 — supporting info [file 41598_2017_1226_MOESM1_ESM.pdf]

SUPPORTING INFORMATION

Aptamer- Based Label-Free Electrochemical Biosensor Array for the Detection of Total and Glycated Hemoglobin in Human Whole Blood

Shimaa Eissa[a], Mohammed Zourob[a,b]*

[a] Department of Chemistry, Alfaisal University, Al Zahrawi Street, Al Maather, Al Takhassusi Road, Riyadh 11533, Saudi Arabia

[b] King Faisal Specialist Hospital and Research Center, Zahrawi Street, Al Maather, Riyadh 12713, Saudi Arabia.

1-Materials and Reagents

The aptamer sequences, the primers for polymerase chain reaction (PCR), the DNA library (5'-ATA TCA TAT GCT CCA ATT-N_{60}-AGATCGCAAGTGTAATAT-3') were synthesized by Integrated DNA Technologies Inc. (Coralville, USA). The thiol modified aptamers are G20: 5′HS−(CH2)6/ GGGGACACAGCAACACACCCACCCACCAGCCCCAGCATCATGCCCATCCGTCGTGT GTG -3′ and G15: 5′HS− (CH2)6/ ACGCACACCAGAGACAAGTAGCCCCCCAAACGCGGCCACGGAACGCAGCACCTCCA TGGC -3′). Ethylenediaminetetraacetic acid, methanol, sodium salt (EDTA), sodium bicarbonate, sodium carbonate anhydrous, sodium azide, disodium dehydrate, boric acid, Tris-base, urea, acrylamide/bis-acrylamide (40% solution) and Taq plus DNA polymerase were purchased from Bioshop Inc. (Ontario, Casnada). TOPO TA Cloning Kit with One Shot MAX Efficiency DH5α-T1, 3,3′,5,5′-tetramethylbenzidine (TMB) stabilized chromogen and HRP-labeled IgG antibody were purchased from Invitrogen (NY, USA). N-hydroxysuccinimide (NHS)-activated Sepharose™ beads, Potassium ferrocyanide ($K_4Fe(CN)_6$), potassium ferricyanide ($K_3Fe(CN)_6$), dipotassium hydrogen orthophosphate, potassium dihydrogen orthophosphate, sodium chloride, magnesium chloride, sulphuric acid, N,N-dimethyl formamide (DMF), cysteamine hydrochloride, pyridine, 1,4-phenylene diisothiocyanate (PDITC), bovine serum albumin (BSA), acetic acid, sodium acetate, potassium nitrate, mercapto-1-hexanol (MCH) and gold (III) chloride (HAuCl4) solution were purchased from Sigma (Ontario, Canada). Purified HbA1c, Hb and anti-human HbA1c antibody were obtained from Monojo (Amman Jordan). The quality control

samples (LN15-08– LN15-11) prepared from pooled whole blood from healthy or diabetic individuals were obtained from College of American Pathologists. Centrifuge desalting Filters (Amicon Ultra-0.5 mL , cut-off: 3kD) were purchased from EMD Millipore (Alberta, Canada). Cellulose acetate centrifuge filters (pore size: 0.45 µm) were obtained from Corning life sciences (Tewksbury MA, USA). The binding buffer composed of 50 mM Tris, pH 7.5, 2 mM $MgCl_2$ and 150 mM NaCl,. Elution buffer is composed of 7 M urea in binding buffer. Tris-EDTA buffer (TE) is 10 mM Tris, pH 7.4, 1 mM EDTA. A 10 mM phosphate buffered saline (PBS) solution (pH 7.4) was used for the ELISA experiments. 0.1 M $NaHCO_3$, 0.5 M NaCl, pH 8.3 was used for the coupling of HbA1c and Hb to the NHS activated sepharose beads. The 1,4-phenylene diisothiocyanate solution was prepared by dissolution in pyridine and N,N-dimethyl formamide (DMF) (v:v, 1:9). All solutions were prepared using Milli-Q grade water.

2-Instrumentation:

Electrochemical experiments were performed using Autolab PGSTAT302N (Eco Chemie, The Netherlands) potentiostat/galvanostat, controlled by Nova 1.11 software. A three-electrode system was used for the binding affinity measurements, consisting of a gold working electrode, Ag/AgCl electrode as the reference and a Pt wire as the auxiliary electrode. The electrochemical biosensing experiments were done using disposable electrical printed (DEP) microarray electrodes from BioDevice Technology (Nomi, Japan). The microarray electrode consists of eight individually addressable carbon working electrodes, a ring-shaped carbon auxiliary electrode, and a central silver/silver chloride reference electrode. A sensor connector (BioDevice Technology) is used to connect the DEP electrodes to the Autolab potentiostat. The UV and fluorescence measurements were performed using NanoDrop 2000C Spectrophotometer and NanoDrop 3300 Fluorospectrometer, respectively (Fisher Scientific, Canada).

3- SELEX protocol for the screening of DNA Aptamers

Three nmol or 1.8×10^{15} sequences of a random ssDNA library were utilised for the screening of the aptamers. Each sequence in the library is composed of a central random region of 60 nucleotides and fixed regions of 18 nucleotides-sequences at the 3' and 5' terminals. The fixed sequences (5'-ATA TCA TAT GCT CCA ATT-N_{60}-AGATCGCAAGTGTAATAT-3') are used as the primers binding sites for the PCR. A hundred µl of HbA1c-beads was washed multiple times with binding buffer and incubated with the pre-treated library. The pre-treatment step of the DNA library was done by heating the DNA solution (3 nmol at the 1st SELEX round and 150 pmol in the subsequent rounds in 300 µL binding buffer) to 90°C for 5 minutes, cooling at 4°C for 10 minutes and incubating it at 25° C for 5 minutes. The beads with the DNA were mixed by rotation in a centrifuge filter tube for 2 hours. After that, the beads were washed extensively with binding buffer. In order to separate the bound DNA to the HbA1c-beads, an elution step was performed by denaturation. Hot Elution buffer was added to the beads (400 µl for 6 times) and heated at 90°C for 10 minutes. The absence of the DNA in the last elution is verified by measuring the fluorescence. The collected DNA solutions from the elution step were then concentrated and desalted using ultrafiltration tube. The counter selection cycle was performed in a similar manner but the DNA solution was first mixed with the negative beads, washed DNA was collected, concentrated and pre-treated by heating and cooling as previously described and subsequently mixed with the HbA1c-beads. Another counter selection round was performed by incubating the DNA pool with Hb-beads and the washed DNA was again collected and incubated with HbA1c-beads. After desalting of the eluted DNA, PCR amplification was performed in 15 parallel 75µL reactions. The PCR mixture is composed of 2 units of Taq Plus and polymerase buffer, 200 µM dNTP, 2 mM $MgCl_2$, 0.2 µM of forward and reverse primers. The PCR reaction conditions are :

94°C for 10 minutes, followed by 25 cycles of 94°C for 1 minute, 47°C for 1 minute, 72°C for 1 minute, and a final extension step of 10 minutes at 72°C. The primers were modified with fluorescein label from one terminus and a PEG linker followed by a poly-A tail from the other terminus as reported previously [19-21]. The PCR primers sequences are : Forward primer: 5'-fluorescein- ATATCATA TGCTCCAATT-3' and reverse primer: 5'- poly-dA$_{20}$-PEG$_{6}$-ATATTACACTTGCGATCT-3'. PCR products were dried by SpeedVac instrument, resuspended in water and formamide (50:50 v/v) and heated to 55°C for 5 minutes. The ssDNA aptamer strand (labelled with fluorescein) was separated from the dsDNA(PCR product) using 12% denaturing PAGE and eluted from the gel band by freeze-thaw cycle in TE buffer. Then, the collected DNA is again concentrated, desalted, quantified by UV and used for the subsequent selection cycle.

4-**Cloning and sequencing of selected DNA**

The cloning of the DNA collected from the last round (round 11 at which the recovery reached almost plateau) is performed by first amplifying the DNA with the non-modified primers set and then cloning into pCR2.1-TOPO vector employing the TOPO TA Cloning Kit. LB-agar medium with ampicillin, X-Gal and IPTG was used to grow the colonies. Then the white colonies were picked and grown in liquid LB media. The ssDNA inserts were then amplified by PCR using the M13 forward and reverse primer sites within the vector and sequenced. The alignment of the selected sequences was done using PRALINE (Figure S4).

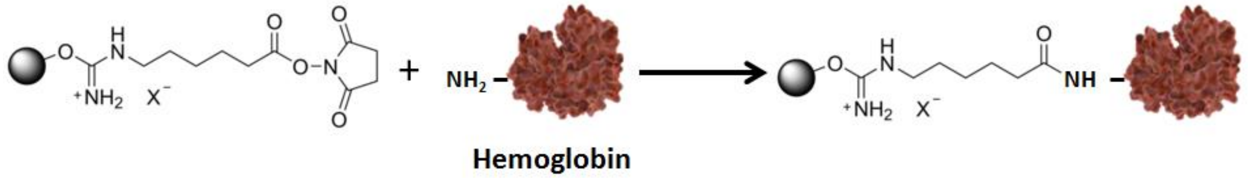

Fig. S1. Attachment of Hb and HbA1C to the NHS-activated beads

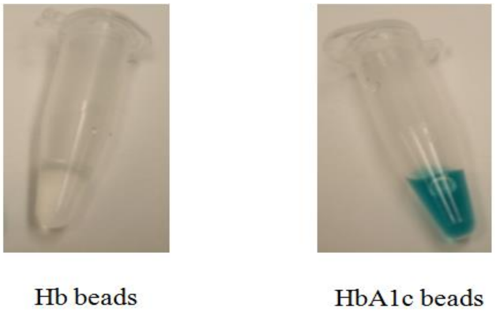

Fig. S2. ELISA results for Hb and HbA1c.

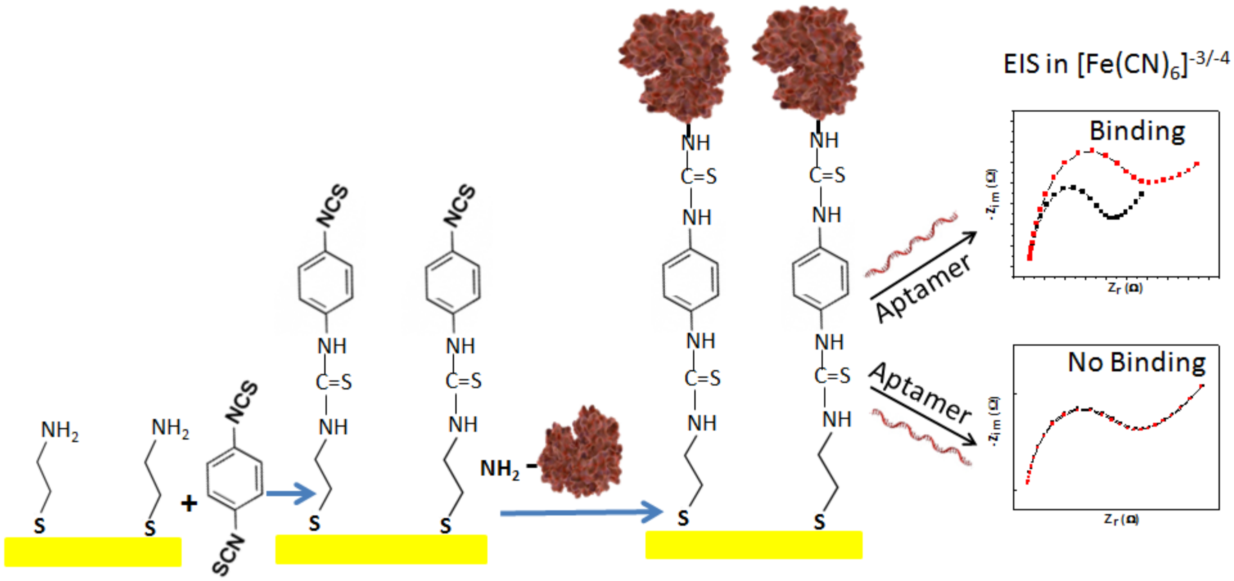

Fig.S3 Schematic diagram of the gold electrode modification and immobilization of Hb and HbA1C proteins for the binding affinity studies.

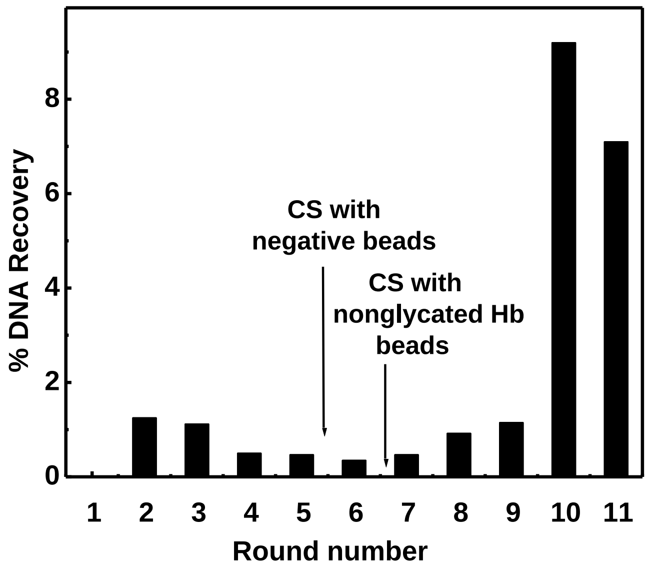

Fig. S4 DNA recovery during the SELEX screening against HbA1c

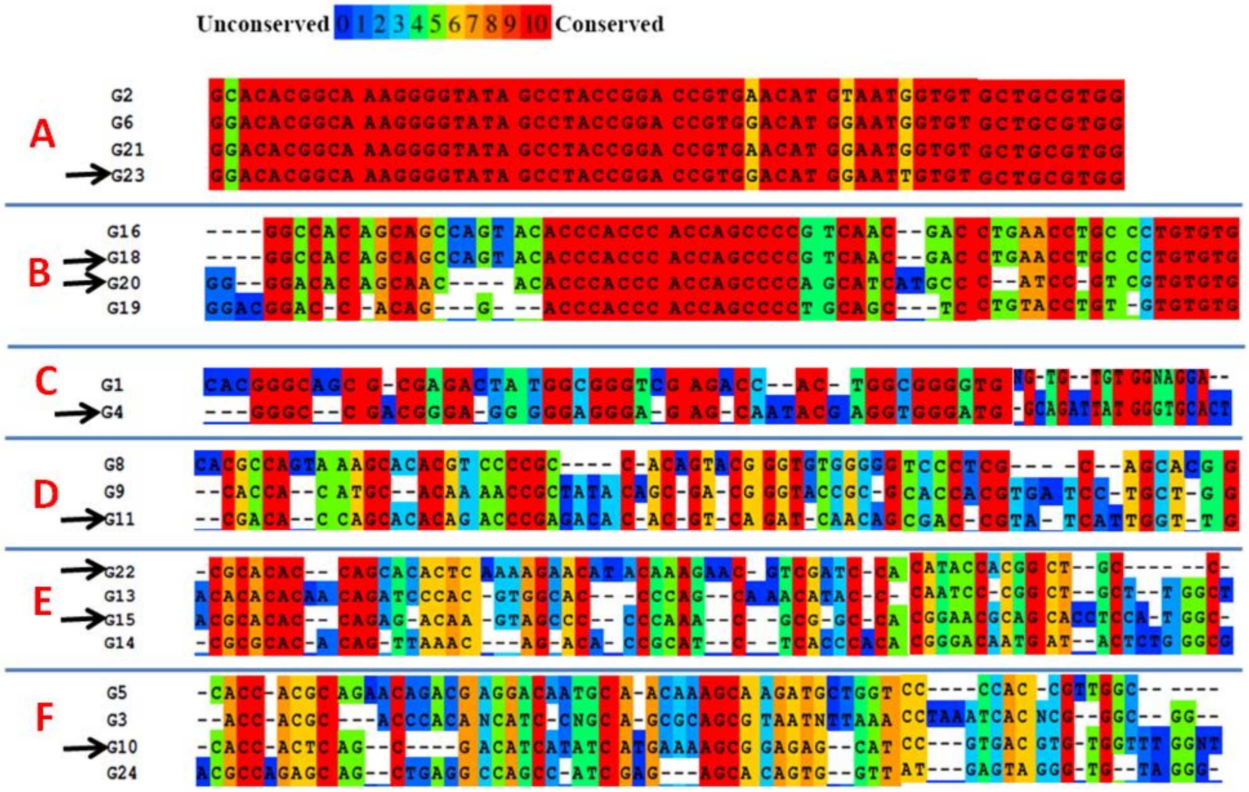

Fig. S5. Multiple sequence alignment of the selected aptamers

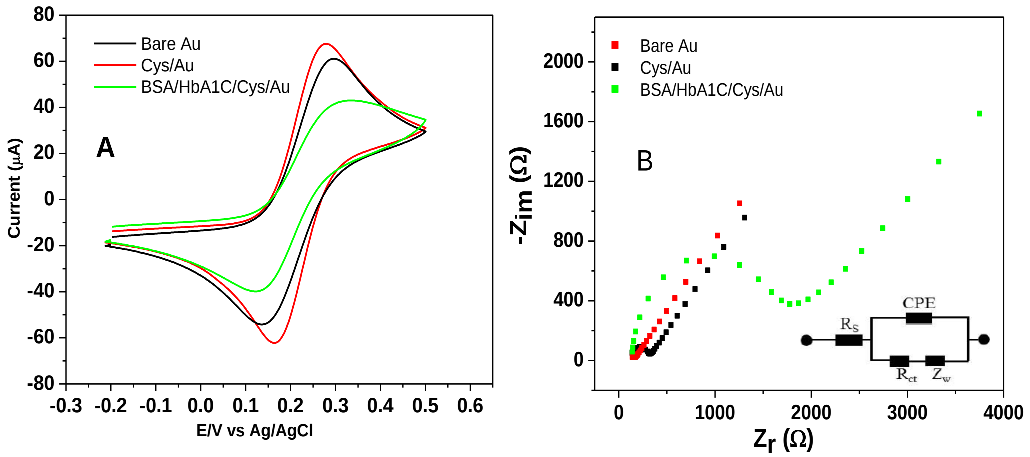

Fig. S6. Cyclic voltammograms (A) and (B) Nyquist diagrams for bare gold electrodes (black),Cys/Au (red) and after HbA1C immobilization and blocking with BSA (green), recorded in 10 mM $[Fe(CN)_6]^{4-/3-}$ redox couple solution in PBS, pH 7.4. The CV was performed at scan rate of 100 mV/s and the EIS was done over the frequency range from 10^5 to 0.1 Hz. The inset is the Randles equivalent circuit applied to fit the EIS.

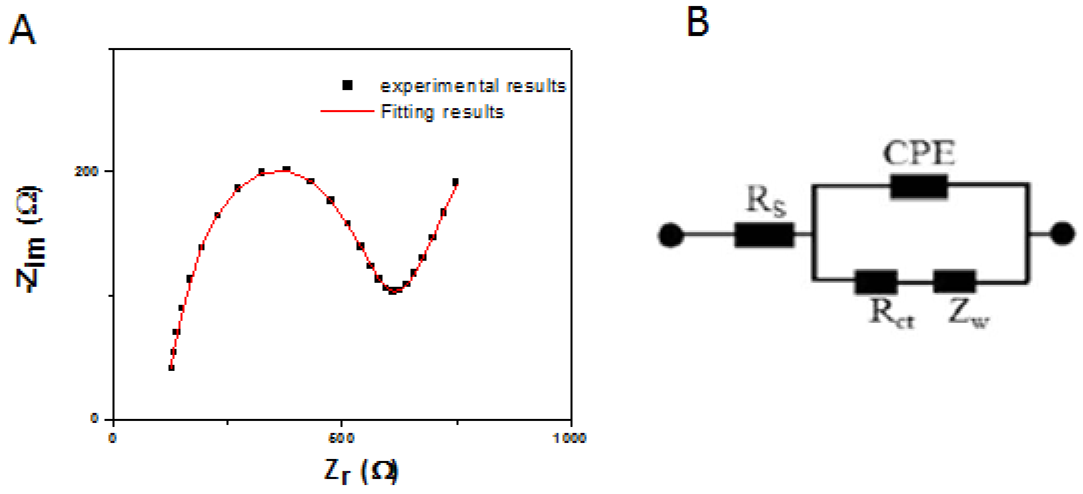

Fig. S7. (A) Example of Nyquist plot. The symbols represent the experimental data and the red curve represents the fitted data using the equivalent circuit. The goodness-of-fit ($\chi 2$) was less than 0.001 for all the impedance results which suggests a good fit between the theoretical and the experimental data. (B) Modified Randles equivalent circuit that was used for fitting. This circuit composed of the solution resistance, R_S, a charge-transfer resistance, R_{CT}, the Warburg impedance, Z_W, representing the diffusion of the redox couple from the bulk of the solution to the electrode interface, and a CPE, representing the electrical double layer capacitance.

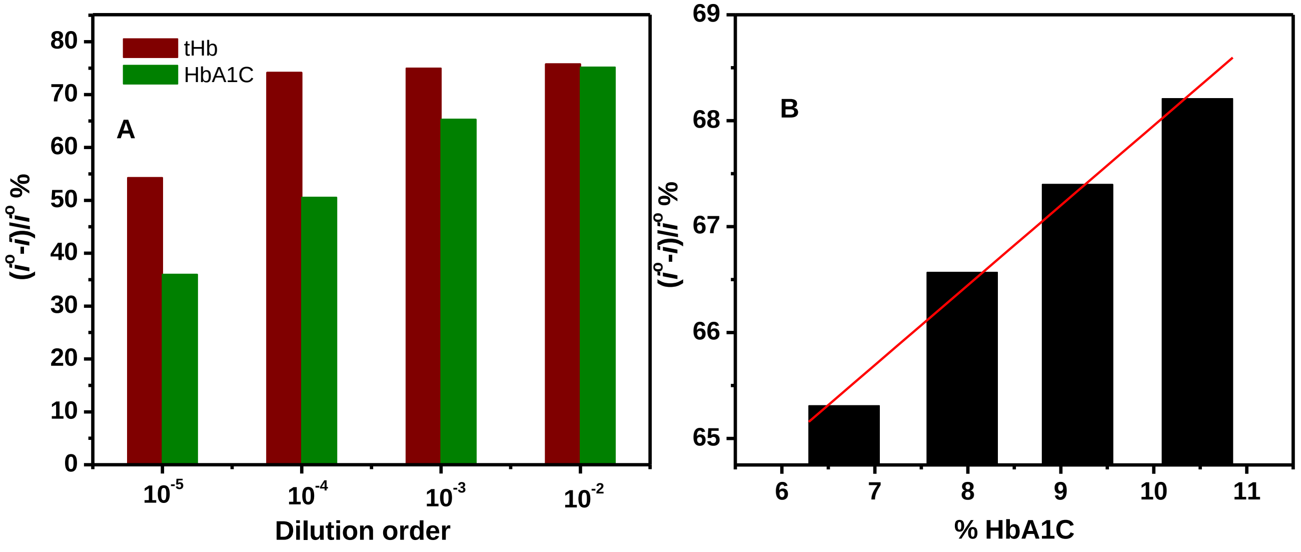

Fig. S8 (A) Response signals of HbA1c and Hb aptasensors for human blood sample (LN15-08) and (B) linear plot of the sensor response for the HbA1c with four different standard blood samples.

Table S1 Comparison of the analytical performance of the developed aptasensor and the other assays

Method	Limit of detection	Analytical range	Reference
Sandwich Immunoassays on Microarrays	3.58 ng/ml for tHb and 0.20 ng/ml for GHbA1c	3.58 - 3.58 µg/ml and 0.20 µg/ml for GHbA1c	[1]
Commercial ELISA	-	10 to 100 ng/ml for tHb and 15.6− 1000 µg/ml for the GHbA1c	Bethyl for Hb and Cusabio Biotech for HbA1c
Commercial ELISA	-	3.125 ng/ml - 200 ng/ml For HbA1c	Abbexa Ltd
Boronic acid-based electrochemical biosensor	-	2.5% to 15% HbA1c per total hemoglobin	[2]
Chemiluminescence aptamer/immunoassay		4.0–14.5% for HbA1c	[3]
Electrochemical Aptasensor	0.34 ng/ml for tHb and 0.20 ng/ml for GHbA1c	100 pg/ml to 100 ng/ml for both tHb and HbA1c	This work

References

1. Chen, H.-H., Wu, C.-H., Tsai, M.-L., Huang, Y.-J. & Chen, S.-H. Detection of Total and A1c-Glycosylated Hemoglobin in Human Whole Blood Using Sandwich Immunoassays on Polydimethylsiloxane-Based Antibody Microarrays. *Analytical Chemistry* **84**, 8635-8641 (2012).
2. Song, S.Y. & Yoon, H.C. Boronic acid-modified thin film interface for specific binding of glycated hemoglobin (HbA1c) and electrochemical biosensing. *Sensors and Actuators B: Chemical* **140**, 233-239 (2009).
3. Lin, H.-I., *et al.* Selection of aptamers specific for glycated hemoglobin and total hemoglobin using on-chip SELEX. *Lab on a Chip* **15**, 486-494 (2015).
